# cGAS- Stimulator of Interferon Genes Signaling in Central Nervous System Disorders

**DOI:** 10.14336/AD.2021.0304

**Published:** 2021-10-01

**Authors:** Fengjuan Li, Ningqun Wang, Yangmin Zheng, Yumin Luo, Yongbo Zhang

**Affiliations:** ^1^Department of Neurology, Beijing Friendship Hospital, Capital Medical University, Beijing 100050, China; ^2^Institute of Cerebrovascular Disease Research and Department of Neurology, Xuanwu Hospital of Capital Medical University, Beijing 100053, China

**Keywords:** cGAS, STING, cGAS-STING, CNS disorders

## Abstract

Cytosolic nucleic acid sensors contribute to the initiation of innate immune responses by playing a critical role in the detection of pathogens and endogenous nucleic acids. The cytosolic DNA sensor cyclic-GMP-AMP synthase (cGAS) and its downstream effector, stimulator of interferon genes (STING), mediate innate immune signaling by promoting the release of type I interferons (IFNs) and other inflammatory cytokines. These biomolecules are suggested to play critical roles in host defense, senescence, and tumor immunity. Recent studies have demonstrated that cGAS-STING signaling is strongly implicated in the pathogenesis of central nervous system (CNS) diseases which are underscored by neuroinflammatory-driven disease progression. Understanding and regulating the interactions between cGAS-STING signaling and the nervous system may thus provide an effective approach to prevent or delay late-onset CNS disorders. Here, we present a review of recent advances in the literature on cGAS-STING signaling and provide a comprehensive overview of the modulatory patterns of the cGAS-STING pathway in CNS disorders.

The innate immune system is the first line of defense against microbial infections and is essential for the activation of adaptive immunity. Innate immune recognition is mediated by a vast array of germline-encoded innate immune receptors, often referred to as pattern recognition receptors (PRRs) [1]. PRRs play an essential role in the sensing of pathogen-associated molecular patterns (PAMPs) and damage-associated molecular patterns (DAMPs). For example, toll-like receptors (TLRs) recognize a variety of PAMPs and DAMPs which initiate the process of inflammation via the activation of nuclear factor (NF)-κB and the synthesis and release of cytokines and interferons (IFNs) [2]. Inflammasomes are a distinct class of intracellularly expressed PRRs that recognize nucleic acids and mediate pro-inflammatory responses [3]. In addition to these PRRs, the cyclic-GMP-AMP synthase-stimulator of interferon genes (cGAS-STING) axis has been identified as a major nucleic acid recognition pathway. cGAS typically resides as an inactive protein in the cell and is activated upon binding to aberrant DNA. Activated cGAS then synthesizes 2′,3′-cGAMP, which acts as a secondary messenger that activates STING [4,5]. Activated STING translocates to the Golgi and activates tank-binding kinase 1 (TBK1), resulting in phosphorylation of TBK1. TBK1 phosphorylates type I interferon regulatory factor 3 (IRF3) [6], which then dimerizes and translocates into the nucleus, where it functions concomitantly with NF-κB, a transcription factor activated by STING. This induces the expression of type I IFNs and inflammatory cytokines, leading to antiviral immune responses [7, 8] (Fig.1).

**Figure 1.**
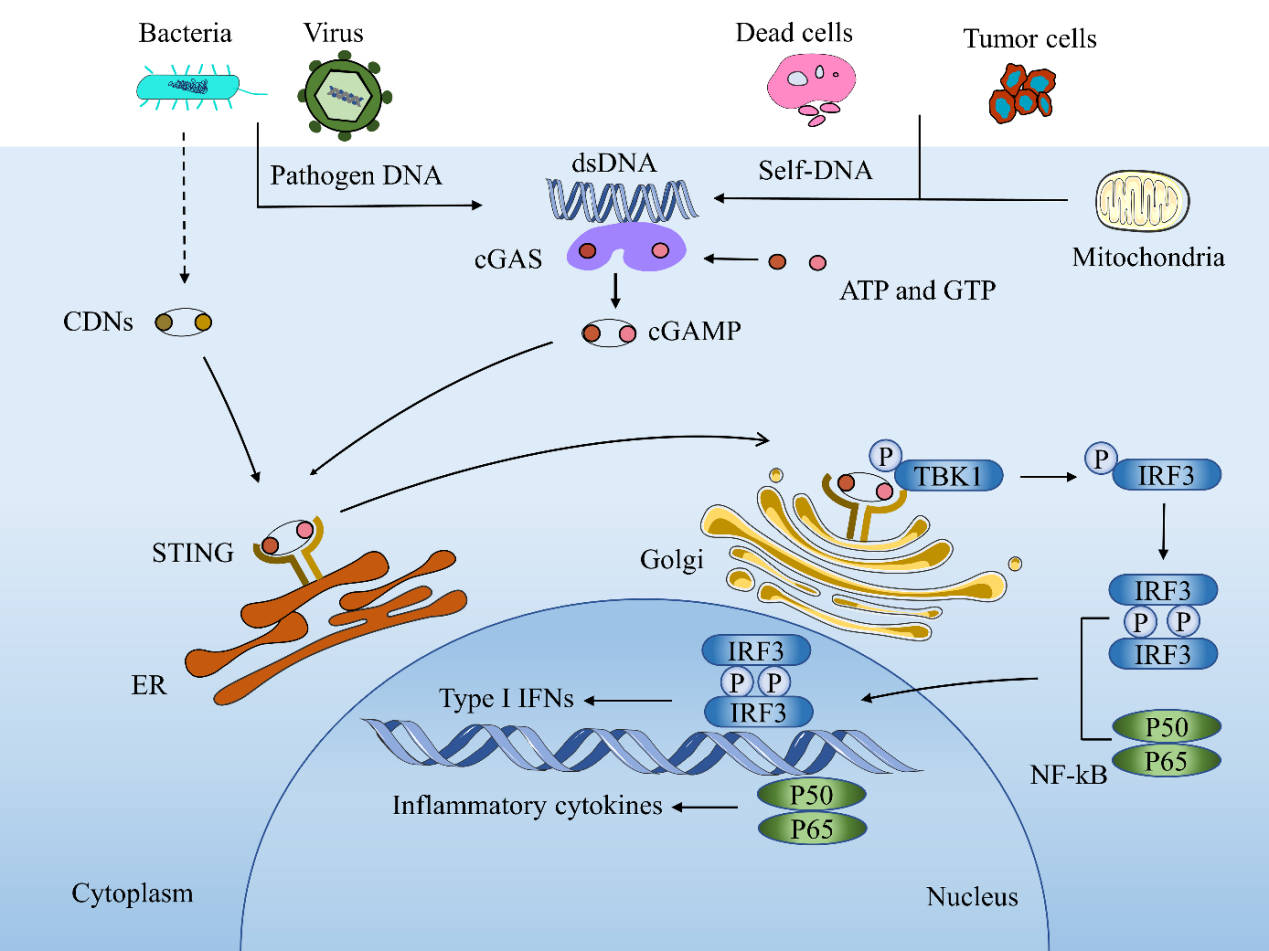
Activation of cGAS-STING signaling pathway. cGAS is activated by sensing cytosolic DNA either from pathogenic DNA or self-DNA. Activated cGAS utilizes ATP and GTP to produce the second messenger cGAMP. cGAMP binds to the ER adaptor STING, which can also be activated by CDNs derived from bacteria. Activated STING translocates from the ER to Golgi compartments and recruits TBK1, which further recruits IRF3 for phosphorylation and dimerization. The phosphorylated IRF3 dimer then enters the nucleus and functions in concert with NF-κB to induce the expression of type I IFNs and inflammatory cytokines.

Mounting evidence has demonstrated that the physiological and pathological relevance of cGAS and STING extends far beyond “traditional” antiviral immunity [8]. Increased cytosolic DNA levels due to factors such as mitotic stress in cancers, cellular senescence, or autoimmune disorders may lead to cGAS-STING activation and aggravation of pathological progression [9-11]. Research on the role of the cGAS-STING pathway in CNS disorders has grown in recent years. Constitutive and systemic activation of cGAS-STING results in chronic neuroinflammation and neurodegeneration. There has yet to be a comprehensive review of this topic. In this review, we present recent advances in the literature on cGAS-STING signaling, focusing on the contribution of the cGAS-STING axis to CNS disorders.

## Recognition of dsDNA by cGAS and formation of cGAMP

cGAS mediates DNA-sensing via direct binding, which triggers conformational changes that induce enzymatic activity [12]. cGAS dimerization increases with DNA binding depending on the length of the bound DNA [13]. Double-stranded DNA (dsDNA) equal to or more than 36bp in length is optimal for recognition by cGAS [4]. cGAS is activated by pathogenic DNA, such as viral and bacterial DNA, as well as self-DNA, such as nuclear DNA derived from dead cells or tumor cells that have damaged DNA repair and mitochondrial DNA (mtDNA) resulting from mitochondrial oxidant damage. Studies have demonstrated that BAX and BAK can permeabilize the outer mitochondrial membrane. In the context of caspase inhibition, these pores grow substantially, allowing inner membrane herniation and extrusion of mtDNA [14, 15]. In the absence of apoptotic caspase, mtDNA activates cGAS in a promiscuous manner, which leads to elevated IFN-β [16, 17]. cGAS was originally assumed to be primarily cytosolic, thereby avoiding persistent activation by self-DNA in the nucleus [18, 19]. The DNA replication and repair factors, RPA and Rad51, constitute an intrinsic cellular mechanism that protects the cytosol from self-DNA [20]. However, this idea has been challenged by several recent studies demonstrating that cGAS is also localized in the nucleus and is tightly tethered to chromatin [21,22]. cGAS has been reported to interact with histone 2A-histone 2B and is tightly anchored to the acidic patch [23-27]. Volkmann et al. demonstrated that the majority of cGAS proteins resided in the nucleus, and the authors proposed a model whereby cGAS must be “desequestered” prior to its full activation [21]. Another study demonstrated that cytosolic cGAS was largely localized to the plasma membrane, which enabled more rapid and efficient detection of viral DNA that entered the cell via endocytosis [28]. Nevertheless, the mechanisms by which cGAS avoids inappropriate sensing of self-DNA remain unclear.

Active cGAS converts GTP and ATP into cGAMP, which contains one 2′,5′- phosphodiester linkage and a canonical 3′,5′-linkage (c[G (2′,5′)pA(3′,5′)p]) [5, 20, 29]. cGAMP activates STING, which triggers type I IFNs responses. Other cyclic dinucleotides (CDNs), including cyclic di-GMP and cyclic di-AMP, are secreted during intracellular bacterial infections and directly activate STING [30, 31]. STING is also known to bind dsDNA directly [32], although the physiological relevance of this remains to be clarified. cGAMP can be transferred between cells via gap junctions, which may stimulate the activation of the IFN pathway in uninfected neighboring cells to promote resistance to infection [33]. cGAMP packaged into viral particles may also be transferred into newly infected cells [34]. SLC19A1, a folate-organic phosphate antiporter, has been implicated in the transport of extracellular cGAMP into the cytosol [35, 36]. Moreover, LRRC8A:C/E transports cGAMP into bystander cells, a process mediated by STING activation [37, 38]. cGAMP is degraded by a specific mammalian phosphodiesterase, ENPP1, which controls cGAMP uptake by cells [39]. In addition to triggering STING, extracellular cGAMP can directly bind to cGAS and induce its activation [40].

### Activation of STING and downstream signals

STING is retained in the endoplasmic reticulum (ER) by interacting with the Ca2+ sensor, stromal interaction molecule 1 (STIM1) [41]. The cytosolic ligand-binding domain (LBD) of STING is the most functional unit capable of integrating with cGAMP. Upon interaction, closure of the ligand binding pocket in the LBD occurs, leading to the activation of STING [42]. Following stimulation, STING traffics to the Golgi and ER-Golgi intermediate compartments (ERGIC), resulting in recruitment of TBK1 and activation of the STING signalsome [43]. STING ER exit protein (STEEP//CxORF56) interacts with STING and promotes trafficking from the ER [44]. This process is mediated by the stimulation of phosphatidylinositol-3-phosphate (PtdIns(3)P) production and ER membrane curvature formation, which induce coat protein complex II (COP-II)-mediated ER-to-Golgi trafficking of STING [44]. SNX8 recruits VPS34 to STING, which is required for trafficking of STING [45]. Various factors, including iRhom2, SCAP, INSIG1, and TMED2 facilitate ER-to-Golgi trafficking [46-49]. Blocking ER-to-Golgi trafficking with brefeldin A and Shigella effector protein IpaJ abolishes phosphorylation of IRF3 and induction of type I IFNs [43, 50, 51]. Further, knockdown of the small GTPase Sar1 regulates COP-II-mediated ER-to-Golgi trafficking and inhibits the translocation of STING from the ER and phosphorylation of IRF3 [52]. Mutations in *COPA*, which encodes the α-subunit of the COPI complex, result in chronic elevation of type I IFNs [53]. COPI promotes retrograde Golgi-ER transport, and mutant *COPA* is associated with an accumulation of STING in the Golgi [54]. These results imply that translocation of STING is associated with its activation. However, the molecular hierarchy of this process and the coordination with COPII trafficking are not fully understood. In the Golgi, STING is palmitoylated at two cysteine residues (Cys88/91), which is necessary for STING activation [55, 56]. The STING signalosome produces a scaffold for the phosphorylation of IRF3 and NF-κB, which further translocate into the nucleus and promote the transcription of genes encoding type I IFNs and other cytokines such as tumor necrosis factor (TNF), interleukin (IL)-1β, and IL-6, which stimulate the immune response [57].

### Regulation of cGAS-STING pathway

Tight regulation of the cGAS-STING pathway is necessary to maintain innate immune homeostasis. Post-translational modifications (PTMs) such as phosphorylation, ubiquitination, and glutamylation play important roles in the regulation of the cGAS-STING pathway. Here, we canvass factors in the literature that may regulate cGAS (Table 1) and STING (Table 2). cGAS and STING are also targeted by various viral proteins, but these lie outside the scope of this review and will not be discussed further.

**Table 1:** The regulation factors of cGAS.

Mechanisms	Factors	Functions	Effects	Ref.
Acetylation	KAT5	Acetylating at multiple lysine residues in its N-terminal domain	Promotes its DNA binding ability	[58]
Ubiquitination	RNF185	K27-linked ubiquitination at K137, K384	Enhances production of IFN	[59]
	TRIM56	Monoubiquitination at K33	Increases its DNA binding activity and cGAMP production	[60]
	TRIM14	Recruiting USP14 to cleave K48-linked ubiquitination at K414	Inhibits its degradation	[61]
Phosphorylation	AKT	Phosphorylation at S305 (human) or S291(mouse)	Inhibits its catalytic activity	[62]
SUMOylation	TRIM38	Sumoylation at K217 or K464	Promotes its stabilization	[63]
	SENP2	Desumoylation at K217 or K464	Induces its degradation	[63]
	SENP7	Desumeylating cGAS	Activates sumoylated cGAS	[64]
Glutamylation	TTL6/TTL4	Polyglutamylation at E272/monoglutamylation at E302	Inhibits its DNA binding capacity	[65]
	CCP6/ CCP5	Deglutamylation at E272/E302	Releases the inhibitory effects of glutamylation	[65]
Other mechanisms	G3BP1	Promoting the formation of large cGAS complexes	Efficient activation of cGAS	[66]
	OASL	Bounding to cGAS	Inhibits its enzyme activity	[67]
	ZCCHC3	Enhancing the binding of cGAS to dsDNA	Efficient innate immune response	[68]

### cGAS-STING signaling in CNS disorders

**Figure 2.**
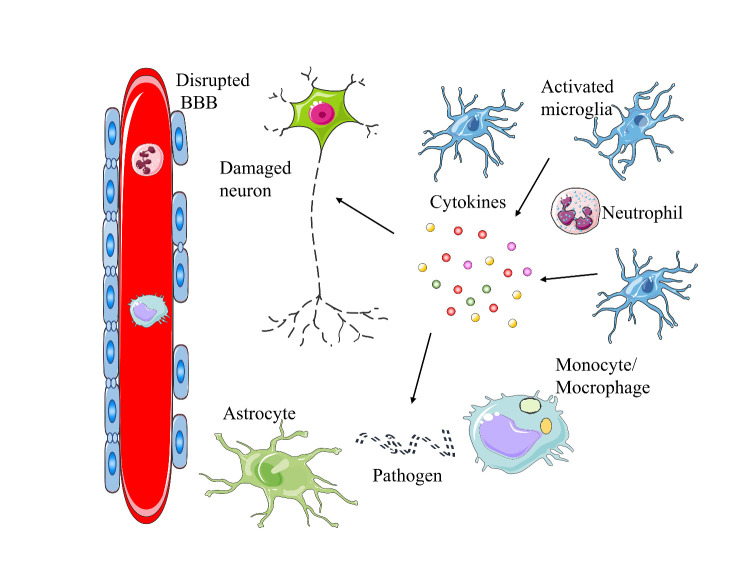
The effect of neuroinflammation in the disruption of CNS homeostasis. Neuroinflammation is accompanied by increased blood-brain barrier (BBB) permeability. Peripheral immune cells, including neutrophils and monocytes/macrophages, are recruited to the lesion site via the disrupted BBB. Microglia are the principal innate immune cells in the brain and produce a range of cytokines at the early stage of neuroinflammation that mediate clearance of pathogens and debris and promote injury repair. In contrast, prolonged neuroinflammation elicits secondary injury, which affects nearby neuronal and glial cells and leads to neurodegeneration.

Neuroinflammation is a CNS defense mechanism induced by various pathological insults such as ischemia, trauma, infection, and toxins [91]. This inflammatory response protects the brain by removing or inhibiting pathogens and promoting tissue repair. However, prolonged neuroinflammation elicits secondary injury, leading to progressive neurodegeneration [92, 93]. The specialized immune system of the CNS detects foreign pathogens and tissue damage, initiates immunological interventions at the local level, and recruits help from the periphery to aid in efficient clearance of pathogens and/or debris [94]. Neuroinflammation leads to infiltration of peripheral immune cells, especially neutrophils and monocytes/macrophages, via the disrupted blood-brain barrier (BBB). Neuroinflammatory responses are mediated by pro-inflammatory cytokines, including IL-1β, IL-6, and TNF; chemokines such as CCL1, CCL5, CXCL1; small-molecule messengers, including nitric oxide (NO) and prostaglandins; and reactive oxygen species (ROS) produced by innate immune cells in the CNS [95]. Microglia are the principal innate immune cells in the brain and the first responders to pathological insults [96]. Indeed, much of the innate immune capacity of the CNS is mediated by microglia [97]. Activated microglia rapidly alter their transcriptional profile, migrate towards sites of injury or infection, and produce inflammatory cytokines and chemokines [94] (Fig.2).

**Figure 3.**
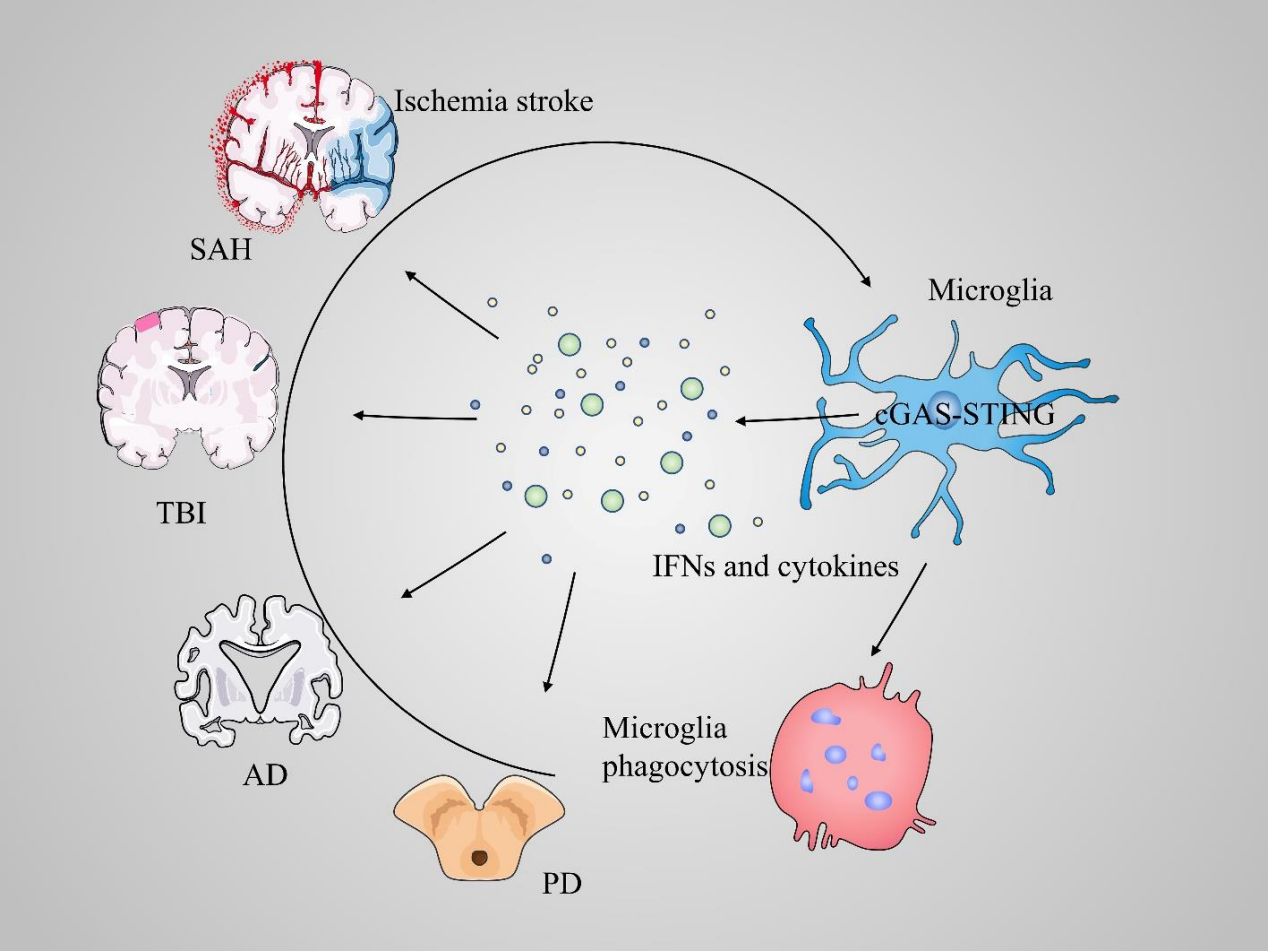
cGAS-STING signaling pathway in CNS disorders. cGAS-STING signaling pathway is involved in neuroinflammation in various CNS disorders such as ischemic stroke, SAH, TBI, AD, and PD. These markers are expressed predominantly in microglia and play different roles depending on the type of disease. This axis acts as a contributing factor to the production of type I IFNs and inflammatory cytokines and promotes microglial phagocytosis. It may also lead to secondary injury and aggravate the pathological progression of CNS disorders.

Activation of the innate immune system involves the induction of the type I IFN-stimulated genes (ISGs) by the mechanisms described earlier. STING is predominantly expressed in microglia, although neurons and astrocytes also produce IFN [95]. Many neuroinflammatory diseases, such as ischemic injury, subarachnoid hemorrhage, traumatic brain injury (TBI), Alzheimer’s disease (AD), and Parkinson’s disease (PD) are characterized by activation of the cGAS-STING pathway and expression of type I IFNs and inflammatory cytokines which underscore pathological progression. In the following text, we discuss the role of cGAS-STING signaling in CNS disorders (Fig.3).

### Ischemic stroke and subarachnoid hemorrhage (SAH)

The cGAS-STING pathway is activated during ischemic injury, which is a debilitating neurological disorder that results in elevated neuroinflammation. Middle cerebral artery occlusion (MCAO), a murine model of ischemic stroke, increases the release of dsDNA into the cytosol and initiates inflammatory responses by activating cGAS [98]. Treatment with A151, an inhibitor of cGAS, reduces the expression of cGAS and neuroinflammatory responses. Moreover, A151 administration significantly reduces infarct volume and ameliorates neurodegeneration in MCAO mice [98]. Liao et al. demonstrated that the microglial cGAS-STING pathway was activated following transient MCAO (tMCAO), which promoted the formation of a pro-inflammatory microenvironment. In addition, they demonstrated that histone deacetylase (HDAC)3 regulated the acetylation and nuclear localization of p65, which promoted the expression of cGAS and potentiated the activation of the cGAS-STING pathway. Deletion of cGAS or HDAC3 in microglia attenuates neuroinflammation and brain injury in tMCAO mice, highlighting a novel therapeutic avenue for the treatment of ischemic stroke [99]. Ischemic stroke is characterized by lack of oxygen and glucose in local brain tissue [100]. McDonough et al. reported that the expression of ISGs was upregulated within microglia exposed to ischemia/reperfusion (I/R) in both *in vitro* and *in vivo* experimental paradigms [101]. Deletion of either IFN-alpha receptor 1 (IFNAR1) or IRF3 exerts protective effects on tMCAO [102, 103]. The STING pathway is also relevant to neovascularization and vascular remodeling. STING knockdown and IFN receptor-neutralizing antibody treatment reduce BBB breakdown and increase vascular plasticity [104]. Collectively, these studies indicate that activation of the endogenous cGAS-STING cascade may be detrimental to the outcomes of ischemic stroke.

Neuroinflammation has recently been implicated in secondary injury following SAH [105]. Preclinical studies have indicated that suppressing neuroinflammation confers increased neurological outcomes after SAH [106, 107]. STING expression increases significantly after SAH, predominantly in microglia. Administration of C-176, a small-molecule inhibitor of STING, confers robust anti-inflammatory effects, alleviates neuroinflammation, and ameliorates short-term and persistent neurological dysfunction after SAH. Further, administration of the STING agonist CMA promotes microglial activation, aggravates neuroinflammation, exacerbates neuronal injury, and increases neurological impairments [108]. These findings suggest that STING is an important regulator of SAH-induced neuroinflammation.

**Table 2:** The regulation factors of STING.

Mechanisms	Factors	Functions	Effects	Ref.
Ubiquitination	TRIM56	K63-linked polyubiquitination at K150	Induces STING dimerization	[69]
	TRIM32	K63-linked polyubiquitination at K20, K224, K236	Promotes the interaction with TBK1	[70]
	MUL1	K63-linked ubiquitination at K224	Enhances IRF3-dependent signaling	[71]
	AMFR	K27-linked polyubiquitination at K137, K150, K224, and K236	Facilitates TBK1 recruitment and activation	[48]
	TRIM30α	K48-linked ubiquitination at K275	Promotes the degradation of STING	[72]
	RNF5	K48-linked polyubiquitination at K150	Mediates its degradation	[73]
	TRIM29	K48-linked polyubiquitination at K370	Mediates its degradation	[74]
	RNF26	K11-linked polyubiquitination at K150	Protects STING from degradation	[75]
	MYSM1	Cleaving K63-linked ubiquitination	Represses the production of IFN	[76]
	USP49	Deconjugating K63-linked ubiquitination	Terminates innate antiviral responses	[77]
	USP20	Deconjugating K48-linked ubiquitination	Facilitates STING-mediated signaling	[78]
	CYLD	Deconjugating K48-linked polyubiquitination	Boosts the innate antiviral response	[79]
	USP21	Hydrolyzing K27/63-linked polyubiquitin chain	Reduces the production of IFN	[80]
	USP13	Deconjugating K27-linked polyubiquitination	Negatively regulates cellular antiviral responses	[81]
	EIF3S5	Deconjugating K48-linked polyubiquitination	Stabilizes STING protein	[46]
SUMOylation	TRIM38	Sumoylating at K337 (murine) or K338 (human)	Promotes its stability and activation	[63]
	SENP2	Desumoylating STING	Induces its degradation	[63]
Phosphorylation	ULK1	Phosphorylating at S366	Suppresses IRF3 activation	[51]
	PTPN1/2	Dephosphorylating at Y245	Leads to its degradation	[82]
	PPM1A	Dephosphorylating at S358	Prevents its aggregation	[83]
	S6K1	Interacting with phosphorylated STING and TBK1	Induces IRF3 activation	[84]
Other mechanisms	TOLLIP	Directing interaction with STING	Prevents STING degradation at the resting state	[85]
	NLRC3	Breaking the association of STING with TBK1	Negatively regulates the innate immune signaling	[86]
	NLRX1	Blocking the assembly of the STING-TBK1 complex	Inhibits IFN response and facilitates viral spread	[87,88]
	ZDHHC1	Mediating STING aggregation and recruitment of TBK1 and IRF3	Positively regulates the innate immune response	[89]
	TMEM203	Interacting with STING	Activates TBK1 and IRF3	[90]
	TMED2	Reinforcing STING dimerization	Potentiates IFN responses	[49]

### Traumatic brain injury (TBI)

TBI is a widespread public health concern that results from excessive contact in sports, blast injuries in war, or occupational hazards [109]. Neuroinflammation plays an integral role in the pathophysiology of TBI by promoting the clearance of debris and regeneration, as well as mediating neuronal death and progressive neurodegeneration [110]. Microglial and peripheral inflammatory cells respond to TBI and provide neuroprotection or participate in maladaptive secondary injury reactions [111]. Type I IFNs are upregulated in postmortem human TBI brains and activate proinflammatory microglia in murine models of TBI [112]. A recent study documented that TBI resulted in acute (within 72 h post-injury) upregulation of cGAS and STING in a mouse model of TBI [113]. Type I IFNs, neuroinflammatory genes, and proinflammatory mediators in the cortex and hippocampus are upregulated following TBI. Knock-down of IFN-β results in decreased levels of these inflammatory markers, and an attenuation of behavioral deficits [113]. Abdullah et al. reported that STING expression was elevated in postmortem human TBI brains; this finding has been confirmed in murine models of TBI. STING deletion suppresses the expression of type I IFNs, accompanied by a reduction in lesion volume [114]. Sen et al. reported that STING signaling was activated by neuronal ER stress [115]. Phosphorylation of protein kinase R-like endoplasmic reticulum kinase (PERK) initiates ER stress after TBI [116]. Blockade of PERK abrogates the STING signaling cascade, thereby reducing neuroinflammation and cognitive impairments [115]. Collectively, these findings highlight a novel targetable signaling axis following TBI.

### Alzheimer’s disease and ataxia-telangiectasia

Alzheimer’s disease (AD) is a chronic neurodegenerative disorder characterized by progressive memory loss and behavioral changes [117]. The essential roles of inflammation in AD pathophysiology is increasingly being recognized [118]. Increased levels of inflammatory markers in patients with AD and the identification of AD risk genes suggest that neuroinflammation plays a prominent role in AD pathogenesis [91, 119]. Indeed, the contribution of neuroinflammation to AD pathogenesis is commensurate (or even exceeds) that of senile plaques and neurofibrillary tangles [120]. Microglial activation was observed at the pre-plaque stage in a transgenic rat model of AD and in individuals with mild cognitive impairment (MCI) without amyloid tracer uptake in a neuroimaging study [121, 122]. Studies have also demonstrated the influence of neuroinflammation at the symptomatic stage of AD [123, 124]. Emerging evidence suggests that the STING pathway is hyperactivated with aging due to internal factors such as chromatin and mtDNA fragments in the cytosol [125, 126]. A rare mutation of the triggering receptor expressed on myeloid cells 2 (TREM2) increases the risk of AD to a similar extent as that for apolipoprotein E (ApoE) ε4 [127, 128]. TREM2 is highly expressed by microglia and promotes Aβ clearance [129]. However, TREM2 mutations aggravate the accumulation of Aβ and neuroinflammation in the brain. Xu et al. reported that cGAMP induced TREM2 expression, which decreased Aβ deposition and ameliorated cognitive impairments [130], highlighting the therapeutic potential of targeting cGAMP to treat AD.

Ataxia-telangiectasia (A-T) is a progressive neurodegenerative disease caused by mutations in the ataxia telangiectasia mutated (*ATM*) gene. ATM plays a major role in sensing and coordinating the repair of DNA double-strand breaks (DSBs). ATM deficiency leads to a breakdown of DNA repair mehanisms and an accumulation of cytoplasmic fragments of nuclear DNA, resulting in activation of the STING signaling cascade and overproduction of cytokines [131]. Inhibition of STING blocks the overproduction of neurotoxic cytokines. ATM deficiency induces STING-mediated IFN production, which promotes anti-microbial immunity [132]. Thus, inhibition of ATM may be a promising approach to boost cellular innate immunity and enhance immune checkpoint blockade therapy. A recent study reported that ATM inhibition potently activated the cGAS-STING pathway and further enhanced immunotherapy by downregulating mitochondrial transcription factor A (TFAM), which resulted in mtDNA leakage into the cytoplasm [133]. Accumulation of cytosolic DNA has been observed in the hippocampus, cerebellum, and spinal cord in rat models of A-T; these events contribute to microglial activation and increased production of IFN-β and IL-1β [134, 135]. Betamethasone treatment reduces neuroinflammatory responses and motor neuron loss, and extends the lifespan of ATM knockout rats [134]. In sum, these studies indicate that cGAS-STING signaling and neuro-inflammation play an essential role in the pathogenesis of AD and A-T.

### Parkinson’s disease (PD), Huntington disease (HD), and amyotrophic lateral sclerosis (ALS)

PD is the second most common age-related neurodegenerative disorder characterized by the progressive loss of dopaminergic neurons in the substantia nigra, involving both motor and non-motor symptoms [136]. Mutations in the leucine-rich repeat kinase 2 (*LRRK2*) gene are a major cause of PD. *LRRK2* is involved in immune system responses and mitochondrial function. Loss of LRRK2 in macrophages induces elevated IFN and ISGs, which are driven by mtDNA leakage into the cytosol and chronic cGAS engagement [137]. Pink1 and Parkin work in concert in mitophagy, thereby removing damaged mitochondria [138]. In a mouse model of *Parkin* knockout mutants combined with a mtDNA mutator strain, selective degeneration of nigral dopaminergic neurons, increased mitochondrial dysfunction, and a decline in motor ability were noted [139]. Supporting these observations, mice lacking *Parkin* and *Pink*1 in mutator combination exhibit a strong inflammatory phenotype that results from mtDNA mutational stress, which activates the cGAS-STING pathway. In addition, genetic inactivation of STING prevents exercise and cytokine production, resulting in rescue of neurodegeneration and locomotor deficits [140]. These findings strongly implicate the induction of STING in the pathogenesis of PD. In contrast, a study reported that loss of STING was insufficient to suppress behavioral deficits or mitochondrial disruption in Drosophila *Pink1/Parkin* or mtDNA mutator models [141]. The reasons for these discrepant results are unclear. One possibility is that aberrant innate immune activation is not mediated by the presence of cytosolic DNA or by activation of the STING pathway. A recent clinical study reported elevated levels of IL-6 and mtDNA in carriers of *Parkin/Pink1* mutations, suggesting that inflammation plays a role in PD pathogenesis [142].

HD is an autosomal dominant inherited neurodegenerative disorder caused by mutations in the Huntingtin gene. HD is characterized by impairments in motor, psychiatric, and cognitive functions [143]. Inflammatory responses are implicated in the pathogenesis of HD [144, 145]. Ribosome profiling revealed that cGAS mRNA has high ribosome occupancy in HD striatal cells derived from mouse embryos. cGAS activity is enhanced, and the expression of inflammatory genes and autophagy proteins is increased. Depletion of cGAS decreases inflammatory and autophagy responses in HD striatal cells, indicating that cGAS promotes inflammatory responses in HD and may be a therapeutic target for HD [146].

ALS is a devastating disease that involves loss of motor neurons, leading to progressive impairments in motor function [147]. In a German ALS population study, higher education and living in a rural environment was associated with a higher risk of developing ALS [148]. In addition, emotional ability at disease onset is associated with faster disease progression in ALS [149]. TAR DNA-binding protein of 43 kDa (TDP-43) is an RNA/DNA-binding protein that regulates mRNA splicing, stability, and translation in the nucleus. Cytoplasmic accumulation of TDP-43 is observed in neurons of almost all patients with ALS [147]. TDP-43-mediated neurodegeneration in ALS is associated with increased proinflammatory cytokine production related to elevated NF-κB and type I IFNs signature [150, 151]; these effects are driven by the cGAS-STING pathway [152]. TDP-43 invades mitochondria and releases mtDNA, which is detected by cGAS and leads to further activation of STING. Further, elevated levels of cGAMP, the specific cGAS signaling metabolite, have been observed in spinal cord samples of ALS patients [152]. Expansions of a GGGGCC repeat in the *C9orf72* gene are the most commonly identified genetic cause of ALS/frontotemporal dementia (C9-ALS/FTD) [153]. Marked expression of type I IFNs mediated by STING in dendritic cells isolated from *C9orf72*-deficient mice have been observed, and blocking STING suppresses the type I IFNs response. Moreover, an elevated type I IFNs signature has been observed in blood-derived macrophages and brain tissue of patients with C9-ALS/FTD, and this elevated signature can be suppressed with administration of a STING inhibitor [154]. Collectively, these findings suggest that targeting the cGAS-STING pathway is a viable therapeutic strategy to alleviate the damage caused by ALS.

### Multiple sclerosis and Aicardi-Goutières syndrome

Multiple sclerosis (MS) is an inflammatory and autoimmune neurological disorder characterized by demyelination [155]. Immunomodulatory therapies such as IFN and rituximab prevent or delay the progression of MS [156]. IFN-β is thought to mediate beneficial effects by targeting innate and adaptive immune cells. Microglia are abundant in MS lesions. Microglial activation is often remote from lesions and may represent the earliest stage of lesion development [157]. Mathur and colleagues reported that ganciclovir (GCV) treatment resulted in an upregulation of several antiviral proteins in cultured microglia, including CXCL10 and IFN-β, at both the mRNA and protein levels [158]. In experimental autoimmune encephalomyelitis (EAE), a mouse model of MS, STING is exclusively expressed in microglia, and GCV induces a type I IFNs response dependent on activated STING. Notably, this response is necessary for GCV to inhibit inflammation in cultured myeloid cells and in EAE. Inhibition of STING pathway mediators, such as STING, IRF3, and TBK1, results in reduced activity of GCV. GCV may mimic CDNs and activate the STING pathway [158]. Similarly, Lemos et al. demonstrated that administration of DNA nanoparticles (DNPs) and CDNs significantly delayed EAE onset and reduced disease severity. DNPs and CDN activates the STING pathway and attenuate infiltration of effector T cells into the CNS, highlighting the beneficial effects of STING *in vivo* [159]. Further, STING/IFN-β is downregulated in relapse-remitting MS (RRMS) patients [160]. These observations have shed insight into the role of STING as a potent immune regulator in MS.

Aicardi-Goutières syndrome (AGS) is a rare lupus-like autoimmune disease characterized by excessive production of type I IFNs. AGS is driven by mutations in genes involved in nucleic acid transactions, including *TREX1, RNASEH2A, RNASEH2B, RNASEH2C, SAMHD1, ADAR1, and IFIH1* [161]. TREX1 (DNase III) is an exonuclease that degrades DNA in the cytoplasm. Loss of Trex1 in dendritic cells is sufficient to cause IFN release and autoimmunity [162]. Genetic ablation of cGAS alleviates autoimmune phenotypes, suppresses the expression of ISGs, and decreases T-cell activation, suggesting that cGAS activation induced by accumulated DNA is involved in AGS [9]. Vincent et al. developed a small-molecule inhibitor of cGAS, RU.521, that reduced constitutive expression of IFN in macrophages in a mouse model of AGS [163]. STING and TBK1 have also been implicated in the inflammatory response of AGS [164, 165]. RNase H2-deficient mice exhibit increased expression of ISGs dependent on the cGAS-STING signaling pathway, and ablation of STING partially rescues perinatal lethality [166]. SAMHD1 is a dNTPase that promotes the degradation of nascent DNA at stalled replication forks in human cell lines. In SAMHD1-depleted cells, the cGAS-STING pathway is activated and induces the expression of IFN [167]. Immunoreactivity in AGS may be underscored by the accumulation of nucleic acids and involvement of the cGAS-STING pathway.

### Encephalitis

Acute viral encephalitis is a devastating disease that can cause irreversible damage and even death [168]. Herpes simplex virus type 1 (HSV-1) is the primary cause of viral encephalitis that accounts for 50-75% of all viral cases [169]. Early production of IFN is critical for controlling the spread of CNS viral infections. Microglia are the main producers of type I IFNs following HSV-1 infection, a response that is dependent on the cGAS-STING signaling axis. Mice defective in cGAS or STING are highly susceptible to HSV-1 infection [170]. HSV-infected microglia confer STING-dependent antiviral activity in neurons and prime type I IFNs production in astrocytes via the TLR3 pathway [170]. HSV-1 infected microglia undergo apoptosis at high viral levels and induce IFN-mediated responses at low viral doses, effects that are dependent on cGAS [171]. Bodda et al. reported that a HSV1 mutant lacking deubiquitinase (DUB) activity of the VP1-2 protein induced elevated IFN expression in microglia and STING phosphorylation [172]. VP1-2 is directly associated with STING, leading to its deubiquitination, blocking IFN expression, and promoting brain infection. DUB activity of HSV1 VP1-2 is a major viral immune-evasion mechanism in the brain [172]. Further, HSV-1 UL37 tegument protein impairs the catalytic ability of cGAS and disarms host defenses. Consistent with these findings, inactivating mutations in HSV-1 UL37 induce more robust cytokine responses, lower brain viral loads, and higher survival rates [173].

Japanese encephalitis virus (JEV), a flavivirus with single-stranded RNA, is recognized by RIG-I and acts in concert with STING to induce IFN-mediated responses. STING ablation inhibits inflammatory molecules and increases intracellular viral load. Conversely, overexpression of STING decreases intracellular viral load [174]. STING signaling is also involved in encephalitis caused by West Nile virus infections, and STING knockouts exhibit increased mortality, viral load, and aberrant T cell responses that are linked with CNS pathology in a murine model of infection [175].

Zika virus (ZIKV) is a member of the flavivirus genus of RNA viruses which can infect the fetal brain during pregnancy and result in significant brain abnormalities [176]. ZIKV predominantly infects neural stem cells and induces serious neurological complications during fetal development [177]. STING confers protection against ZIKV by inducing autophagy, while loss of autophagy leads to increased ZIKV infection and death [178]. Cao et al. reported that inhibition of autophagy limited vertical transmission of ZIKV and ameliorated adverse placental or fetal outcomes in a mouse model of pregnancy [179]. ZIKV attenuates STING signaling in primate cells via NS2B3 protease [180]. Lennemann and Coyne demonstrated that ZIKV NS2B3 protease cleaved FAM134B (an ER-localized reticulophagy receptor), suppressed the reticulophagy pathway, and promoted viral replication [181]. Nevertheless, it remains unclear whether autophagy suppresses ZIKV infections in the mammalian brain.

### Conclusions

The cGAS-STING pathway is essential for modulation of the innate immune response. This signaling pathway is a double-?edged sword in CNS disorders. It acts as a contributing factor by providing defense mechanisms via the regulation of type I IFNs production and spreading of immune signaling to adjacent cells. However, its overactivation may lead to secondary injury and aggravate the pathological progression of CNS disorders. These findings have spurred efforts to harness this natural defense-related pathway in the generation of brain disorders therapy.

In this review, we summarize the recent advances in cGAS-STING signaling, and its crucial role in the pathogenesis of CNS disorders. The activation of cGAS and STING may exert either positive or negative influences, depending on the context. Further research on this pathway will pave the way for deriving novel targets to halt disease progression or reverse symptoms at an early stage. For example, remaining questions include how the different regulators of the cGAS-STING pathway reciprocally interact and how the activities of these enzymes are regulated in a timely manner. To date, studies of the cGAS-STING pathway in CNS disorders have predominantly been conducted in preclinical settings, and further studies are necessary to explore the clinical relevance of this pathway.
